# Malnutrition Decreases Antibody Secreting Cell Numbers Induced by an Oral Attenuated Human Rotavirus Vaccine in a Human Infant Fecal Microbiota Transplanted Gnotobiotic Pig Model

**DOI:** 10.3389/fimmu.2020.00196

**Published:** 2020-02-14

**Authors:** Husheem Michael, Stephanie N. Langel, Ayako Miyazaki, Francine C. Paim, Juliet Chepngeno, Moyasar A. Alhamo, David D. Fischer, Vishal Srivastava, Dipak Kathayat, Loic Deblais, Gireesh Rajashekara, Linda J. Saif, Anastasia N. Vlasova

**Affiliations:** ^1^Food Animal Health Research Program, Department of Veterinary Preventive Medicine, Ohio Agricultural Research and Development Center, The Ohio State University, Wooster, OH, United States; ^2^Division of Viral Disease and Epidemiology, National Institute of Animal Health, National Agriculture and Food Research Organization, Tsukuba, Japan

**Keywords:** rotavirus, vaccine, malnutrition, adaptive immunity, B cells, human microbiota

## Abstract

Human rotavirus (HRV) is a leading cause of morbidity and mortality in children, especially in developing countries. Malnutrition is prevalent in these countries, which may contribute to the decreased oral vaccine efficacy, posing a concern for global health. Neonatal gnotobiotic (Gn) pigs closely resemble human infants in their anatomy, physiology, and outbred status and are a unique model to investigate malnutrition, oral live attenuated HRV (AttHRV) vaccination, and subsequent virulent HRV (VirHRV) challenge. We evaluated the impact of malnutrition on AttHRV vaccine efficacy and B cell immune responses in neonatal germfree (GF) or Gn pigs transplanted with human infant fecal microbiota (HIFM). Pigs were fed either deficient or sufficient bovine milk diets. Malnutrition did not significantly affect the serum and intestinal contents total or HRV-specific IgG and IgA antibody titers pre VirHRV challenge. However, HRV-specific IgG and IgA antibody secreting cells (ASCs) were reduced in blood or intestinal tissues following AttHRV vaccination and pre VirHRV challenge in deficient HIFM transplanted pigs. Furthermore, post-VirHRV challenge, deficient HIFM pigs had decreased total Ig and HRV-specific IgG and IgA antibody titers in serum or intestinal contents, in addition to decreased HRV-specific IgG and IgA ASCs in blood and ileum, compared with sufficient HIFM pigs. Our results indicate that deficient diet impairs B cell mucosal, and systemic immune responses following HRV vaccination, and challenge. The impaired immune responses contributed to the decreased protective efficacy of the AttHRV vaccine, suggesting that malnutrition may significantly reduce the effectiveness of oral HRV vaccines in children in developing countries.

## Introduction

Human rotavirus (HRV) is a leading cause of diarrhea in children. It causes significant morbidity and mortality, especially in developing countries (1). Additionally, the efficacy of RV vaccines is low in developing compared with developed countries (2). Malnutrition, micronutrient deficiencies, and breast milk antibodies are implicated in the lower efficacy of RV vaccines (3–5). Malnutrition is a major contributor to the high mortality from viral gastroenteritis in low socio-economic status countries (6–8). Some clinical studies have reported decreased protection rates against RV diarrhea following RV vaccination of malnourished children (9, 10). A number of field and animal studies have shown that malnutrition induces immune dysfunction, including altered innate and adaptive immune responses, impairment of epithelial cell barriers and dysfunction of intestinal epithelial stem cells (11–16). However, studies to elucidate the effect of malnutrition on B cell responses following oral HRV vaccine are lacking.

Recently Miyazaki et al. have shown that protein deficiency impaired multiple aspects of innate, T cell, and cytokine immune responses that resulted in reduced efficacy of an oral live attenuated (AttHRV) vaccine in neonatal gnotobiotic (Gn) pigs. In addition, protein deficient HIFM pigs showed prolonged and higher titers of virus shedding following virulent HRV (VirHRV) challenge, suggesting that transplanted human infant fecal microbiota (HIFM) exacerbated the negative impact of protein deficiency on HRV T cell immunity and virus infection (17). We have shown previously that protein deficiency resulted in suppression of multiple innate immune parameters involved in the dendritic cell-IL12-natural killer cell immune axis that play an important role in regulating VirHRV infection (11). Further we observed that suppression of this immune axis following oral AttHRV vaccination in deficient HIFM pigs, resulted in failure to promote subsequent innate and T cell immunity. In addition, serum levels of kynurenine (a metabolite of the amino acid tryptophan that promotes immunosuppression in response to inflammation) were reduced in deficient HIFM pigs. Hence, the impaired vaccine efficacy and immune responses observed in the deficient HIFM pigs could be attributed to the impaired innate, pDCs, and activated CD4^+^ T cell responses and the altered cytokine responses following AttHRV vaccination and VirHRV challenge.

Gnotobiotic (Gn) pigs are immunocompetent at birth, but immunologically immature (18). As in humans, secretory IgA is dominant in the intestine, milk, and mucosal secretions (19). HRV infected Gn pigs exhibit diarrhea, transient viremia and intestinal lesions mimicking those in children (20). Gn pigs are caesarian-derived and housed in sterile isolators to assure their germfree status, permitting studies of gut colonization with single bacteria or transplantation with the complete fecal microbiota. Thus, Gn pigs are a unique model to study direct and indirect effects of malnutrition on host metabolism, neonatal immune responses, enteric viral infections or oral vaccines without other confounding microbiota (19, 21). Importantly, transplantation of HIFM into Gn piglets recapitulates the infant microbial community (11, 22). The resulting microbiota humanized Gn pigs allow manipulation of multiple variables that are not possible in infants or conventional animal models and the sampling of gut responses.

Previously we have established a protein deficient HIFM-transplanted neonatal Gn pig model that recapitulates major aspects of protein malnutrition in children (11, 12). Because malnutrition is prevalent in African and South Asian countries with low HRV vaccine efficacy, we hypothesized that malnutrition will affect the B cell immune responses to oral AttHRV vaccination and subsequent VirHRV challenge. B cells play an important role in response to malnutrition and in generation of protective antibodies against RV infection (23, 24).

To further understand the immunological and biologic mechanisms underlying the reduced RV vaccine efficacy, our goal was to investigate B cell immune responses after AttHRV vaccination and VirHRV challenge in the HIFM transplanted Gn pigs that were fed deficient or -sufficient diets. We also included a non-HIFM transplanted GF pig counterpart group to elucidate the immunomodulating effects of the transplanted HIFM on malnutrition and on the other study parameters.

## Materials and Methods

### Human Infant Fecal Microbiota (HIFM)

The collection and use of HIFM were approved by The Ohio State University Institutional Review Board. With parental consent, sequential fecal samples were collected from a healthy, 2-month-old, exclusively breastfed, vaginally delivered infant. Samples were pooled and diluted to 1:20 (wt/vol) in PBS containing 0.05% (vol/vol) cysteine and 30% glycerol and stored at −80°C as described previously (11, 12).

### Virus

The cell-culture adapted attenuated HRV (AttHRV) Wa G1P [8] strain passaged in African green monkey kidney cells (MA-104) was used as a vaccine at a dose of 1 × 10^7^ fluorescent foci-forming units (FFU) (25). The Gn pig passaged virulent HRV (VirHRV) Wa strain at pig passage 26 was used as challenge virus at a dose of 1 × 10^6^ FFU as described previously (11, 12).

### Animal Experiments

The animal experiments were approved by the Institutional Animal Care and Use Committee at The Ohio State University. Piglets were derived from near-term sows by hysterectomy and maintained in sterile isolators (26). The detailed experimental design and procedures were described earlier by Miyazaki et al. (17). Briefly, neonatal pigs obtained from five litters (5–15 pigs/litter) were randomly assigned to one of four groups: (1) deficient diet, GF (no HIFM) (Deficient group, *n* = 12); (2) sufficient diet, GF (no HIFM) (Sufficient group, *n* = 11); (3) deficient diet, HIFM transplanted (Deficient HIFM group, *n* = 12); and (4) sufficient diet, HIFM transplanted (Sufficient HIFM group, *n* = 11). Pigs in Sufficient GF and Sufficient HIFM groups were fed 100% ultra-high temperature pasteurized bovine milk (Parmalat) that met or exceeded the National Research Council Animal Care Committee's guidelines for calories, fat, protein and carbohydrates in suckling pigs. Pigs in Deficient GF and Deficient HIFM groups were fed 50% bovine milk diluted with 50% sterile water which contained half of the recommended protein levels (7.5 vs. 15% of diet). All pigs were confirmed as free from bacterial and fungal contamination by aerobic and anaerobic cultures of rectal swabs prior to HIFM transplantation. Pigs in deficient HIFM and Sufficient HIFM groups were orally inoculated with 2 ml of diluted HIFM stock at 4 days of age (post-HIFM transplantation day, PTD 0). Rectal swabs were collected once or twice a week to analyze the microbial composition by 16S metagenomic analysis as described previously (11). All pigs were orally vaccinated twice at a 10-day interval with AttHRV at PTD 7/post-1st vaccination day, PVD 0 and PTD 17/PVD 10 [post-2nd vaccination day 0, thereafter referred as PVD10 (0)]. At PTD 24/PVD 17 (7)/post-challenge day (PCD) 0, a subset of pigs from each of the four groups were euthanized to assess vaccine responses pre-challenge. The remaining pigs were challenged with VirHRV and euthanized at PTD 31/PVD 24 (14)/PCD 7. Serum, intestinal contents, and mononuclear cells samples were collected on the day of euthanasia.

### Isolation of Mononuclear Cells (MNCs)

Blood, duodenum, and ileum were collected to isolate MNCs as described previously (27–29). The tissues were collected aseptically and placed in ice-cold wash medium (RPMI 1640 with 10 mM HEPES, 200 μg/ml gentamicin, and 20 μg/ml ampicillin). Tissues were smashed through stainless steel 80-mesh screens of a cell collector to obtain single-cell suspensions. Segments of duodenum and ileum were rinsed twice each with wash medium and Ca^2+^ and Mg^2+^ free Hanks balanced salt solution (Gibco BRL, Gaithersburg, MD) and mechanically rotated, and vortexed to dislodge epithelial cells, and intraepithelial lymphocytes. After removal of epithelial cells, the segments were minced, suspended in RPMI 1640 containing 8% FBS, 200 μg/ml gentamicin, 20 μg/ml ampicillin, 20 mM HEPES, 5 mM EDTA, 10 mM DTT and 400 U/ml of type II collagenase (Sigma Chemical Co., St. Louis, Mo) and digested for 30 min at 37°C with gentle shaking. The digested supernatants (containing lamina propria lymphocytes) were collected, and the remaining tissues were pressed through 80-mesh screens to obtain single-cell suspensions. The cell suspensions from duodenum, and ileum were suspended in a 90% isotonic percoll solution (Sigma Chemical Co.) and centrifuged at 1,200×g for 30 min at 4°C. Cell pellets were re-suspended in 45% Percoll, underlaid with 70% Percoll, and centrifuged at 1,200× g for 30 min at 4°C. The MNCs were collected from the 45-to-70% interface and washed once with wash medium. Blood was collected in 30% (vol/vol) acid citrate glucose, and peripheral blood lymphocytes (PBL) were obtained by Ficoll-Paque (Sigma Chemical Co.) density gradient centrifugation. Cells at the interface were collected and washed twice in Hanks balanced salt solution. Erythrocytes were lysed with 1 ml sterile water. The purified MNCs were suspended in E-RPMI 1640. The viability of each MNCs preparation was determined by trypan blue exclusion (≥95%).

### Determination of HRV-Specific and Total Antibody Responses

The HRV specific and total antibody titers in serum and intestinal contents were detected as described previously (12, 30–33). To determine the intestinal antibody responses, small intestinal contents were collected with protease inhibitors in the medium. Briefly, 96-well plates (Nunc-Maxisorp) were coated overnight at 4°C with guinea pig hyperimmune serum against group A RV (1:10,000 diluted in coating buffer pH 9.6) for 2 h at 37°C. Subsequently plates were blocked with 2% milk in PBS containing 0.1% tween-20 at 4°C overnight. Semi-purified Wa HRV or mock MA-104 cell supernatants were added to the alternate columns of plates. Serial 4-fold dilutions of each test sample were added to the antigen-coated or mock-coated wells and incubated for an hour and 30 min at 37°C. For HRV IgA, IgM, and IgG antibody detection, goat anti-pig IgA HRP (1:3,000, AA140P, Serotec), goat anti-swine IgM (1:2500, 04-14-03, KPL), and biotinylated goat anti-swine IgG (gamma chain) (1:20000. 16-14-02, KPL) were used, respectively. For detecting IgG isotype, HRP conjugated streptavidine was used to detect HRV specific IgG1 and IgG2 antibodies (1:10,000, Roche, 11089153001). The ELISA antibody titer (geometric mean titers [GMTs]) was expressed as the reciprocal of the highest dilution at the corrected OD value at 405 nm (sample OD in the virus-coated well minus sample OD in the mock antigen-coated well) greater than the cut-off value (mean raw OD values of the positive capture of the negative samples + 2× standard deviations of the OD values of the positive capture of the negative samples).

For total IgM, IgA, and IgG titer determination, 96-well plates were coated overnight with goat anti-pig IgA (1:16000, A100-102A, Bethyl), –IgM (1:2000, A100-117A, Bethyl), or –IgG (1:8000, A100-105A, Bethyl) polyclonal antibodies. Plates were then blocked with 4% skim milk and incubated for 1 h at 37°C. Pig reference serum was used as standards (RS 10107, Bethyl). Samples or standards were incubated for 1 h at 37°C. For antibody detection, goat anti-pig IgA (1:16000, A100-102P, Bethyl), –IgG (1:8000, 04-14-02, KPL), and -IgM (1:2000, 04-14-03, KPL) were used. TMB substrate was developed and the reaction stopped by adding 1M phosphoric acid and absorbance read at 450 nm.

### Determination of HRV-Specific Antibody Secreting Cell (ASC) and Total Ig Secreting Cell (IgSC) Responses

ELISPOT assay for HRV-specific and total IgSCs in blood, duodenum, and ileum were conducted and ASCs and IgSCs were enumerated as described previously (30–33). ELISPOT for HRV-specific ASCs: Briefly, confluent monolayers of MA104 cells in 96-well plates (Nunc-Immuno) were inoculated with 100 μl of cell culture-passaged Wa rotavirus per well (1.25 × 10^6^ FFU/ml) containing 0.05% trypsin. After incubation for 14 h at 37°C in 5% CO_2_, the cells were then fixed with 70% acetone for 15 min. The plates were air dried and were stored at −20°C until further use. Acetone fixed plates were thawed, hydrated with RPMI 1640 and the 100 μl MNCs from various tissues were added to plates in duplicate wells (5 × 10^5^, 5 × 10^4^, 5 × 10^3^ or 5 × 10^2^ cells/well). Plates were incubated at 37°C for 12 h in presence of 5% CO_2_. Plates were washed 5 times with PBS-tween 20 (0.05%) and subsequently goat anti-pig IgA HRP (1:3000, AA140P, Serotec), –IgG-biotin (1:20000, 16-14-02, KPL), and –IgM-peroxidase labeled (1:1500, 04-14-03, KPL) antibodies were added and incubated overnight at 4°C. The HRP-conjugated streptavidin was added to the wells that received pig IgG antibody. The plates were washed and spots were developed with a TMB substrate method (KPL) containing membrane developer, and HRV specific antibody secreting cells were counted using a microscope (40×).

Affinity-purified goat anti-pig IgM (50 μg/ml, 01-14-03, KPL), –IgA (30 μg/ml, AA140, Bethyl), and –IgG (30 μg/ml, 01-14-02, KPL) were used as isotype-specific capture antibodies to quantitate total IgSCs in the ELISPOT assay. The capture antibodies were diluted in 50 mM carbonate buffer (pH 9.6), and 100 ml per well was added to 96-well plates (Nunc-Immuno). The plates were incubated for 2 h at 37°C. Plates were then was washed twice with PBS-Tween 20 and blocked with 4% skim milk at 4°C overnight. Each dilution (100 μl) of MNC suspension from various tissues were added to plates in duplicate wells (5 × 10^5^, 5 × 10^4^, 5 × 10^3^ or 5 × 10^2^ cells/well). The plates were incubated for 12 h at 37°C in 5% CO_2_. The plates were then washed and spots were developed as described for HRV-specific ASC.

## Statistical Analysis

All statistical analyses were performed using GraphPad Prism version 7 (GraphPad software, Inc., La Jolla, CA). Log_2_ transformed ELISA isotype antibody titers were analyzed using one-way ANOVA followed by Duncan's multiple range test. Data presented as the mean numbers of HRV-specific antibody secreting cells per 5 × 10^5^ mononuclear cells and statistical differences were considered significant at *P*-values < 0.05 for all comparisons. Error bars indicate the standard error of the mean. To assess a possible linear association between viral shedding/diarrhea scores and one or more immunological parameters, correlation analysis was performed using nonparametric correlation Spearman's method. To compare frequencies of ASCs, IgSCs, as well Ab and Ig titers/concentrations, two-way ANOVA was performed followed by Bonferroni posttest.

## Results

### Malnutrition Affected Generation of HRV-Specific Antibodies in Serum and Intestinal Contents Both After AttHRV Vaccination and Post-VirHRV Challenge

**Figure 1:**
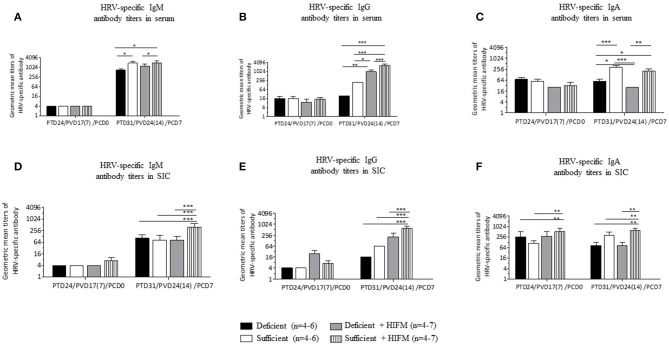
Malnourished pigs had significantly reduced titers of HRV specific IgA antibodies post-VirHRV challenge in serum and small intestinal contents (SIC) compared with sufficient pigs. Geometric mean titers of HRV specific IgM **(A)**, IgG **(B)**, and IgA **(C)** antibodies in serum. HRV specific IgM **(D)**, IgG **(E)**, and IgA **(F)** antibody titers in small intestinal contents. Data is presented as log_2_ and significant differences (*p < 0.05, **p < 0.01, ***p < 0.001) are indicated. Gnotobiotic pigs were transplanted with human infant fecal microbiota (HIFM) at 4 days of age. Pigs were fed deficient diet (Deficient) and sufficient diet (Sufficient). All the pigs were orally vaccinated twice with AttHRV on post transplantation day (PTD) 7 and 17, challenged with virulent human rotavirus (VirHRV) on PTD24/PVD17(7)/PCD0 and euthanized on PTD31/PVD24(14)/PCD7. PVD, post 1st (2nd) vaccination day; PCD, post-challenge day.

No significant differences were observed between deficient and sufficient groups in HRV-specific IgM, IgG, and IgA antibody titers in serum after two AttHRV vaccinations and regardless of HIFM transplantation (Figures 1A–C, **PCD0**). However, deficient pigs had lower titers of HRV-specific IgM and IgG antibodies and significantly lower IgA antibodies in serum post-VirHRV challenge (Figures 1A–C, **PCD7**). In addition, IgG titers were lower in GF pigs compared with HIFM pigs. Similarly, no differences were observed between deficient and sufficient groups in HRV-specific IgM and IgG antibody titers in small intestinal contents pre-challenge (Figures 1D,E, **PCD0**). However, HRV-specific IgA antibody titers were marginally lower in deficient HIFM compared with sufficient HIFM pigs (Figure 1F, **PCD0**). HRV-specific IgM antibody titers were significantly increased in sufficient HIFM pigs compared with deficient HIFM pigs post-VirHRV challenge (Figure 1D, **PCD7**). Deficient HIFM pigs had lower HRV-specific IgG and IgA antibody titers significantly in small intestinal contents post-VirHRV challenge (Figures 1E,F, **PCD7**). Similar trends were observed in GF counterparts. In addition, IgG titers were lower in GF pigs compared with HIFM pigs in small intestinal contents. Moreover, HRV-specific IgA antibody titers in both serum and small intestinal contents were negatively correlated with peak VirHRV shedding titers (*R* = −0.6; *p* = 0.004, and R = −0.4; *p* = 0.05, respectively, Supplementary Table 1). Similarly, HRV-specific IgG and IgA antibody titers in serum were negatively correlated with diarrheal score (*R* = −0.4; *p* = 0.03, and *R* = −0.4; *p* = 0.04, respectively, Supplementary Table 2). These findings indicate that malnourished pigs generated lower HRV-specific antibody responses in serum and small intestinal contents post-VirHRV challenge. In contrast sufficient diet pigs generated significantly increased IgA antibody responses to the AttHRV vaccination, reflected by increased B cell memory IgA antibody responses in serum and small intestinal contents post-VirHRV challenge, and protection against VirHRV challenge that was associated with decreased viral shedding and diarrhea (Table 1) (17).

**Table 1:** Summary of diarrhea and fecal VirHRV shedding after VirHRV challenge (PCD 1—PCD 6).

**Groups^a^**	***N***	**Diarrhea^b^**			**Virus shedding^c^**		
		**%**	**Mean cumulative fecal score^d^**	**Mean duration (days)^e^**	**%**	**Geometric mean of peak titer(FFU ml)^f^**	**Mean duration (days)**
Deficient	6	66.6	6.3	2.5	33.3	157	1
Sufficient	6	50	5.3	0.8	33.3	138	1
Deficient + HIFM	6	33.3	5.3	1.2	100	797.4^**^	2.5
Sufficient + HIFM	6	0	3.8	N/A	33.3	35.4^*^	1.5

a *Gnotobiotic pigs were transplanted with human infant fecal microbiota (HIFM) at 4 days of age. Pigs were fed deficient diet (Def) and sufficient diet (Suf). All the pigs were orally vaccinated twice with AttHRV on post transplantation day (PTD) 7 and 17, challenged with virulent human rotavirus (VirHRV) on PTD24/PVD17(7)/PCDO and euthanized on PTD31/PVD24(14)/PCD7. PVD, post 1st (2nd) vaccination day; PCD, post-challenge day*.

b *Pigs with fecal score > 1 were considered as diarrheic. Fecal consistency was scored as follows: 0, normal; 1, pasty; 2; semi-liquid; and 3, liquid*.

c *Determined by cell culture immunofluorescence assay and expressed as FFU/ml*.

d *Mean of total of fecal score from PCD 1 to PCD7*.

e *Mean of the total days with fecal score > 1*.

f *Samples negative for HRV detection (<25) were assigned a titer of 12.5 for statistical analysis. Means in the same column with different asterisks differ significantly*.

*N/A, data not available*.

### Malnourished Pigs Had Significantly Lower Total Immunoglobulin Concentrations in Serum and Intestinal Contents Post-VirHRV Challenge

**Figure 2:**
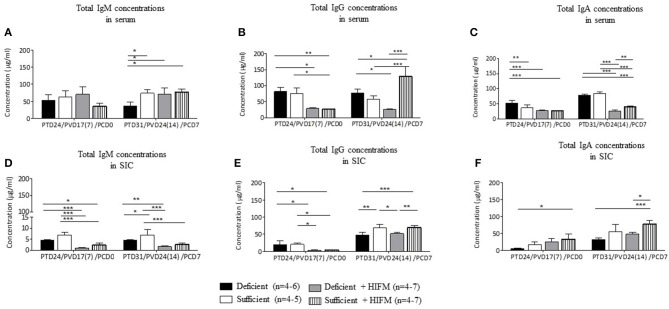
Malnourished pigs had significantly reduced total Ig concentrations in serum and small intestinal contents (SIC) post-VirHRV challenge. Total IgM **(A)**, IgG **(B)**, and IgA **(C)** concentrations in serum. Total IgM **(D)**, IgG **(E)**, and IgA **(F)** concentrations in small intestinal contents. Significant differences (*p < 0.05, **p < 0.01, ***p < 0.001) are indicated. Gnotobiotic pigs were transplanted with human infant fecal microbiota (HIFM) at 4 days of age. Pigs were fed deficient diet (Deficient) and sufficient diet (Sufficient). All the pigs were orally vaccinated twice with AttHRV on post transplantation day (PTD) 7 and 17, challenged with virulent human rotavirus (VirHRV) on PTD24/PVD17(7)/PCD0 and euthanized on PTD31/PVD24(14)/PCD7. PVD, post 1st (2nd) vaccination day; PCD, post-challenge day.

Deficient and sufficient diet pigs did not have significantly altered concentrations of total IgM, IgG, and IgA in serum and small intestinal contents pre-VirHRV challenge (Figures 2A–F, **PCD0**). Following VirHRV challenge, total IgM concentrations were reduced in serum or small intestinal contents, respectively, of deficient vs. sufficient GF pigs, while in HIFM transplanted pigs they were marginally reduced (Figures 2A,D, **PCD7**). Except total IgG concentration in deficient GF, total IgG, and IgA concentrations were reduced significantly or numerically in deficient GF vs. sufficient GF pigs but were significantly lower in deficient HIFM pigs compared with the sufficient counterparts in serum and small intestinal contents (Figures 2B,C,E,F, **PCD7**). The ratios of HRV-specific antibodies to total immunoglobulins were also determined (Supplementary Tables 3, 4).

### Malnutrition Affected Frequencies of HRV-Specific Antibody Secreting Cells (ASCs) in Blood and Intestinal Tissues After AttHRV Vaccination and Post-VirHRV Challenge

**Figure 3:**
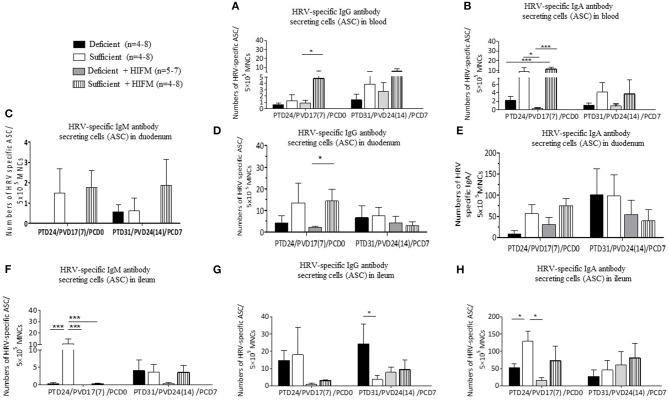
Malnourished pigs had significantly reduced HRV-specific IgG and IgA antibody secreting cells (ASCs) post-AttHRV vaccination and numerically reduced ASC (HIFM pigs) post-VirHRV challenge in blood, duodenum, and ileum. HRV specific IgG **(A)** and IgA **(B)** ASCs in blood. HRV specific IgM **(C)**, IgG **(D)**, and IgA **(E)** ASCs in duodenum. Note 10× difference in y axis scale for C (IgM ASCs). Note 5× difference in y axis scale for E (IgA ASC). HRV specific IgM **(F)**, IgG **(G)**, and IgA **(H)** ASCs in ileum. Note 5× difference in y axis scale for H (IgA ASCs). Significant differences (*p < 0.05, ***p < 0.001) are indicated. Gnotobiotic pigs were transplanted with human infant fecal microbiota (HIFM) at 4 days of age. Pigs were fed deficient diet (Deficient) and sufficient diet (Sufficient). All the pigs were orally vaccinated twice with AttHRV on post transplantation day (PTD) 7 and 17, challenged with virulent human rotavirus (VirHRV) on PTD24/PVD17(7)/PCD0 and euthanized on PTD31/PVD24(14)/PCD7. PVD, post 1st (2nd) vaccination day; PCD, post-challenge day.

Post-AttHRV and pre-challenge, no HRV-specific IgM ASCs were detected in blood. Significantly decreased numbers of HRV-specific IgG and IgA ASCs were generated in the blood of deficient HIFM pigs compared with sufficient counterparts (Figures 3A,B, **PCD0**). Similarly, sufficient HIFM pigs had numerically higher HRV-specific IgG and IgA ASCs in blood compared with deficient pigs post-VirHRV challenge (Figures 3A,B, **PCD7**).

Notably, HRV-specific ASC responses in duodenum, generally more closely paralleled those in blood than in ileum, especially pre-challenge. Pre-VirHRV challenge, increased numbers of HRV-specific IgM and IgG ASCs were detected in the duodenum of sufficient pigs, suggesting that deficient pigs had decreased capacity to respond locally to the AttHRV vaccination (Figures 3C,D, **PCD0**). Mean HRV-specific IgA ASC numbers pre-VirHRV challenge in duodenum and ileum were much higher (6–103-folds) than in blood, but otherwise trends were similar where all sufficient pigs consistently had higher mean numbers of IgA ASCs than deficient pigs (Figures 3B,E,H, **PCD0**). Similarly, except for IgM and IgG ASCs of HIFM sufficient pigs, no significant differences were detected between deficient and sufficient pigs in the numbers of ASCs in duodenum (Figures 3C,D, **PCD7**). Interestingly, post-VirHRV challenge, the opposite trend was seen in duodenum where mean HRV-specific IgA ASC numbers were higher (two fold) in GF than in HIFM pigs (Figure 3E, **PCD7**). Moreover, HRV-specific IgM ASC numbers were negatively correlated with peak VirHRV shedding titers (*R* = −0.4; *p* = 0.04, Supplementary Table 1).

Pre-challenge, mean numbers of HRV-specific IgM ASCs in ileum were numerically higher in GF sufficient compared with HIFM transplanted pigs (Figure 3F, **PCD0**). HRV-specific IgM, IgG and IgA ASCs were slightly decreased in deficient HIFM pigs in ileum post-VirHRV challenge (Figures 3F–H, **PCD7**). Interestingly, significantly higher mean numbers of HRV-specific IgG ASCs were generated in ileum of deficient GF pigs compared with sufficient GF pigs post-VirHRV challenge, with the opposite trend in HIFM pigs (Figure 3G, **PCD7**). Deficient pigs had decreased numbers of HRV-specific IgA ASCs compared with sufficient pigs in ileum both pre- (statistically significant for GF pigs) and post-VirHRV challenge (not significant) (Figure 3H, **PCD0 and PCD7**).

### Malnutrition Affected Generation of Total Immunoglobulin Secreting Cells (IgSCs) in Blood and Intestinal Tissues After AttHRV Vaccination and Post-VirHRV Challenge

**Figure 4:**
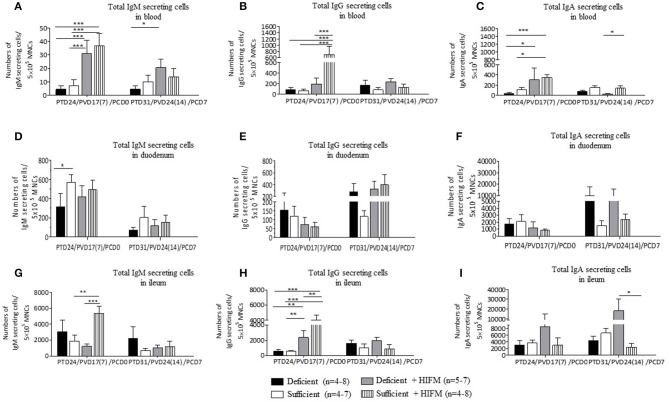
Malnutrition reduced total Ig secreting cells (IgSCs) both post-AttHRV vaccination, pre- and post-VirHRV challenge in blood and ileum. Total IgM **(A)**, IgG **(B)**, and IgA **(C)** IgSCs in blood. Total IgM **(D)**, IgG **(E)**, and IgA **(F)** IgSCs in duodenum. Total IgM **(G)**, IgG **(H)**, and IgA **(I)** IgSCs in ileum. Note difference in y axis scale for IgM compared with IgG and IgA IgSCs in blood and for IgM and IgG compared with IgA IgSCs in duodenum and ileum. Significant differences (*p < 0.05, **p < 0.01, ***p < 0.001) are indicated. Gnotobiotic pigs were transplanted with human infant fecal microbiota (HIFM) at 4 days of age. Pigs were fed deficient diet (Deficient) and sufficient diet (Sufficient). All the pigs were orally vaccinated twice with AttHRV on post transplantation day (PTD) 7 and 17, challenged with virulent human rotavirus (VirHRV) on PTD24/PVD17(7)/PCD0 and euthanized on PTD31/PVD24(14)/PCD7. PVD, post 1st (2nd) vaccination day; PCD, post-challenge day.

Overall the highest total IgM and IgA IgSCs were detected in intestinal tissues compared with blood. Total mean blood IgM IgSCs were increased in HIFM-colonized pigs compared with GF pigs both pre- and post-VirHRV challenge. This could be due to increased local toll-like receptor stimulation in the gut from HIFM leading to increased IgM IgSCs in the blood. Pre-challenge, total IgM IgSCs were numerically decreased (not significantly) in deficient pigs compared with sufficient pigs in blood (Figure 4A, **PCD0**). Post-VirHRV challenge, decreased IgM IgSCs were observed in deficient GF pigs compared to sufficient GF pigs (Figure 4A, **PCD7**), but malnourished HIFM pigs had higher total IgM IgSCs in blood compared with sufficient HIFM pigs.

Malnourished HIFM pigs had significantly lower numbers of total IgG IgSCs in blood compared with sufficient HIFM pigs pre-VirHRV challenge (Figure 4B, **PCD0**). In addition, blood total IgG IgSCs were higher in sufficient HIFM colonized compared with sufficient GF pigs. Post-VirHRV challenge, the mean numbers of total blood IgG IgSCs were consistently higher in deficient pigs, although not significantly (Figure 4B, **PCD7**).

Decreased numbers of total IgA IgSCs were detected in the blood of deficient pigs compared with sufficient pigs pre- and post-VirHRV challenge (Figure 4C). Colonization of both deficient and sufficient pigs with HIFM led to an increased number of total IgA IgSCs pre challenge. Sufficient HIFM pigs had significantly higher total IgA IgSCs compared with deficient HIFM post-VirHRV challenge (Figure 4C, **PCD7**). These results suggest that sufficient pigs had increased B cell responses and IgSCs numbers after HIFM colonization and HRV vaccination/challenge.

Pre- and post-HRV challenge, the mean numbers of total duodenum IgM IgSCs were higher in sufficient pigs (Figure 4D). There were no apparent trends in duodenum total IgG IgSCs except for higher numbers of IgSCs in both deficient GF and HIFM pigs pre- and post-challenge (Figure 4E).

There were no significant differences in total numbers of duodenal IgA IgSCs pre or post-VirHRV challenge. However, deficient pigs had 3-6 folds higher total IgA IgSCs in duodenum compared with sufficient HIFM pigs post-VirHRV (Figure 4F). Similar to the ileum, the increase in HRV shedding titers in deficient compared with sufficient pigs likely stimulated local production of IgA IgSCs (17).

Ileum total IgM IgSCs were significantly higher in sufficient HIFM compared with deficient HIFM pigs pre-challenge (Figure 4G, **PCD0**). However, deficient GF pigs had increased numbers of total IgM IgSCs compared with sufficient GF pigs pre- and post-VirHRV challenge.

Similar to total blood IgG IgSCs, total ileum IgG IgSCs were higher in HIFM colonized compared with GF pigs pre-VirHRV challenge in both deficient and sufficient pigs (Figure 4H, **PCD0**). In addition, sufficient HIFM pigs had significantly increased numbers of total IgG IgSCs compared with deficient HIFM pigs. Post-VirHRV challenge, the mean numbers of blood total IgG IgSCs were numerically higher in deficient treatment groups (Figure 4H, **PCD7**).

No obvious trends were evident for total IgA IgSCs in ileum of GF pigs pre-challenge (Figure 4I, **PCD0**) but deficient HIFM pigs had higher total IgA IgSCs pre- and post-VirHRV challenge compared with sufficient HIFM pigs (Figure 4I, **PCD7**). The increase in HRV shedding titers post-challenge in the deficient HIFM pigs (17) likely stimulated the proliferation of local gut total IgA IgSCs in ileum. The ratios of HRV-specific antibody secreting cells to total immunoglobulin secreting cells were also determined (Supplementary Tables 3, 4).

## Discussion

In this study, we observed that malnutrition affects the B cell antibody responses to HRV that resulted in exacerbation of HRV infection in the HRV challenged, HIFM transplanted neonatal Gn pig model of childhood malnutrition. IgA is the key immunoglobulin in mucosal immunity and is mainly found in mucosal secretions (34). The presence of HRV-specific IgA antibodies in the feces, intestinal contents and serum of humans and pigs are strongly correlated with protection from HRV infection in multiple studies (29, 35–41). Previous research has shown that malnutrition can impair mucosal immunity where IgA plays an important role in host protection and may partly explain the reduced efficacy of oral vaccines against cholera and *Salmonella enterica* serovar *Typhimurium* in the malnourished mice model (42). Moreover, it was shown that malnutrition altered B cell differentiation status in bone marrow, led to a predominance of plasma cells among small intestinal lamina propria lymphocytes, decreased polymeric immunoglobulin receptor expression, and reduced secretory IgA in mucosal sites (43). In addition, malnutrition is known to lead to reversible reduction in IgA antibody responses to antigens encountered at the intestinal mucosa (44). Similarly, in a murine model, it was shown that the enteric pathogens *Cryptosporidium* (45) and entero aggregative *Escherichia coli* (46) exacerbated growth impairment and intestinal damage associated with malnutrition. Thus, malnutrition can be the reason for reduced protective B cell antibody responses to oral vaccines in malnourished children in developing countries.

In this study, malnutrition resulted in decreased HRV-specific IgA and IgG antibodies in serum, intestinal contents, and decreased HRV-specific IgA and IgG ASCs in blood, and intestinal tissues (except HRV-specific IgG and IgA ASCs in duodenum), indicating an impaired mucosal and systemic HRV antibody response following VirHRV challenge. These findings coincide with our prior findings showing impaired innate, T cell, and cytokine immune responses, prolonged diarrhea and higher titers of virus shedding following VirHRV challenge (17). Moreover, our findings are in agreement with previous reports that malnutrition impaired secretory IgA antibody production in humans and mice (23, 43, 44, 47). Additionally, the ratio of total Ig levels and HRV-specific IgG and IgM antibody titers were reduced in serum of the deficient compared with sufficient pigs, further suggesting that malnutrition compromised antibody responses following HRV infection. Interestingly, our investigation showed that malnutrition did not affect the ratio of levels of total Ig and HRV-specific IgG and IgA antibody titers in serum following AttHRV vaccination and pre VirHRV challenge. On the other hand, malnutrition did affect the ratio of levels of total IgSCs and HRV-specific IgG and IgA ASCs in HIFM pigs pre VirHRV challenge in blood and intestinal tissues, that coincided with decreased induction of HRV-specific IgG and IgA antibodies post-VirHRV challenge. The comparisons of microbiota transplantation and diets are shown (Supplementary Table 5). Furthermore, we showed that: (1) the ratio of total IgSCs and HRV-specific IgA and IgG ASCs in blood coincided with protection data post-VirHRV challenge (17); and (2) Sufficient pigs were able to mount a greater ratio of total Ig and HRV-specific antibody response leading to greater protection post-VirHRV challenge. Total IgA IgSCs tended to be higher in deficient HIFM pigs compared with their sufficient counterparts in ileum suggesting increased HIFM stimulation or HRV shedding titers caused local stimulation of total IgA IgSCs. However, mean HRV-specific IgA ASCs were higher in both groups of sufficient pigs than in deficient pigs in ileum. This suggests that deficient piglets had decreased ability to generate HRV-specific antibody responses, which would be more reflective of specific protection post-VirHRV challenge than total IgSCs numbers. Total IgG IgSCs were significantly higher in HIFM pigs compared with GF pigs in ileum suggesting increased local gut stimulation of IgSCs by microbiota in the HIFM pigs. Higher total IgM IgSCs in deficient HIFM compared with deficient GF pigs in ileum also suggested stimulation of IgM IgSCs by microbiota.

Like ileum, total IgA IgSCs tended to be higher in HIFM deficient pigs compared with sufficient pigs in duodenum. However, HRV-specific IgA, IgG, and IgM ASCs all tended to be the highest in sufficient animals compared to deficient pigs. This data suggests that in the duodenum, as seen in ileum, the deficient pigs could not generate an adequate HRV-specific response and overcompensated by generating more non-specific total IgSCs leading to lack of HRV clearance in the gut. This was associated with decreased protective efficacy of the AttHRV vaccine in deficient piglets. Interestingly, this was an opposite trend compared with total IgA IgSCs in blood. For instance, at both pre- and post-VirHRV challenge, sufficient pigs had higher total IgA IgSCs in blood compared to deficient pigs. It is likely that the higher mean total IgA IgSCs in blood of these pigs also reflects increased immunity post-AttHRV vaccination as demonstrated by better protection following VirHRV challenge in sufficient pigs. Moreover, the increase in duodenum mean total IgA IgSCs post-HRV challenge in deficient pigs may represent local stimulation and proliferation of gut IgA ASCs in response to HIFM and HRV.

In malnourished children, it was shown that malnutrition induced altered B cell differentiation status in bone marrow and a predominance of plasma cells in the small intestine lamina propria lymphocytes, decreased pIgR expression, and reduced secretory IgA in mucosal sites, which can be a reason for poor protective immune responses to oral vaccines in malnourished children in developing countries (43). A reduction in the number of IgA plasma cells in the intestinal lamina propria, as seen in malnourished children and rats (48–50), may also be responsible for the reduced numbers of HRV-specific IgA ASCs in blood and intestinal tissues in our investigations. Various investigators have shown a decrease in IgSCs under malnutrition. For instance, (1) The ability of the cells committed to IgA production to home to mucosal tissues could be impaired (51, 52). (2) Lopez et al. (53) found that severely protein-deficient rats exhibited fewer IgM- and IgA-bearing B cells in the Peyer's patches. (3) Barry and Pierce (50) reported the decreased numbers of cholera toxin-specific cells in the thoracic duct of protein deficient rats after oral immunization.

The composition of gut microbiota affects the immune system in different ways (54–57). However, in our investigation we observed mostly similar effects of malnutrition on HRV-specific B cell responses between GF and HIFM pigs (Supplementary Table 5). However, there were exceptions when differences were observed between GF and HIFM pigs: higher HRV-specific IgG antibody titers in deficient HIFM pigs in small intestinal contents pre-challenge; higher total IgG concentrations in deficient GF pigs in serum post-challenge; higher HRV-specific IgG and IgA ASCs in duodenum in GF pigs post-challenge; higher HRV-specific IgG ASCs in ileum post-challenge; higher total IgM IgSCs in deficient HIFM pigs in blood post-challenge; higher total IgG IgSCs in duodenum in deficient GF pigs post-challenge; higher total IgA IgSCs in duodenum in deficient pigs post-challenge; higher total IgM IgSCs in deficient GF pigs in ileum pre- and post-challenge; higher total IgA IgSCs in deficient HIFM pigs in ileum pre- and post-challenge.

Experimental evidence in animal models has shown that B cell antibody responses play a critical role in development of long-lasting protective immunity against RV infection (24, 29). In our future studies, additional analyses such as metabolomics, lipidomics, and transcriptome analysis will help to clarify the underlying mechanisms of the impairment of HRV vaccine immune responses associated with malnutrition and an altered microbiota.

Our findings suggest that impaired B cell immunity of malnourished vaccinated, but unprotected children could result in continuous circulation of RV in the community and co-circulation of genotypically different strains, which increase the risk of RV infections and increased RV diarrhea burden among infants and children in these communities.

In conclusion, using the HIFM transplanted and GF neonatal pig models, we demonstrated that malnutrition impaired the efficacy of an oral AttHRV vaccine by altering B cell antibody responses. Also, the reduced efficacy of the oral AttHRV vaccine was associated with impaired innate, T cell, and cytokine immunity and altered tryptophan- kynurenine catabolic pathway in our prior studies. Furthermore, we found that malnutrition did not affect the total Ig concentrations and HRV-specific IgM, IgG, and IgA antibody titers in serum and intestinal contents following AttHRV vaccination and pre VirHRV challenge. However, it did affect the generation of HRV-specific IgG and IgA ASCs pre VirHRV challenge in blood and intestinal tissues. Our findings of impaired B cell antibody immunity provides novel insights and identifies possible predictors of AttHRV vaccine efficacy that could be beneficial to children in developing countries. There is a growing interest in delineating the interactions among nutrition, metabolites, microbiota and host immunity and the impacts of these interactions on infections and vaccine immunity. The HIFM transplanted neonatal pig model can be further used for mechanistic evaluation of the effects of physiologically relevant interventions.

## Data Availability Statement

The raw data supporting the conclusions of this article will be made available by the authors, without undue reservation, to any qualified researcher.

## Ethics Statement

The animal experiments were approved by the Institutional Animal Care and Use Committee at The Ohio State University.

## Author Contributions

Conceived and designed the experiments: AV, LS, and GR. Data collection: AM, HM, SL, FP, JC, MA, DF, VS, DK, and LD. Analyzed the data: SL and HM. Wrote the paper: HM. Critically revised the paper: AV, LS, and GR.

### Conflict of Interest

The authors declare that the research was conducted in the absence of any commercial or financial relationships that could be construed as a potential conflict of interest.
