# Supplementary material for: Malnutrition Decreases Antibody Secreting Cell Numbers Induced by an Oral Attenuated Human Rotavirus Vaccine in a Human Infant Fecal Microbiota Transplanted Gnotobiotic Pig Model
Source: Front Immunol. 2020 Feb 14;11:196. doi: 10.3389/fimmu.2020.00196 (PMC7033455; doi:10.3389/fimmu.2020.00196)
Supplement: Supplementary file 1 [file Presentation_1.PPTX]

## Slide 1
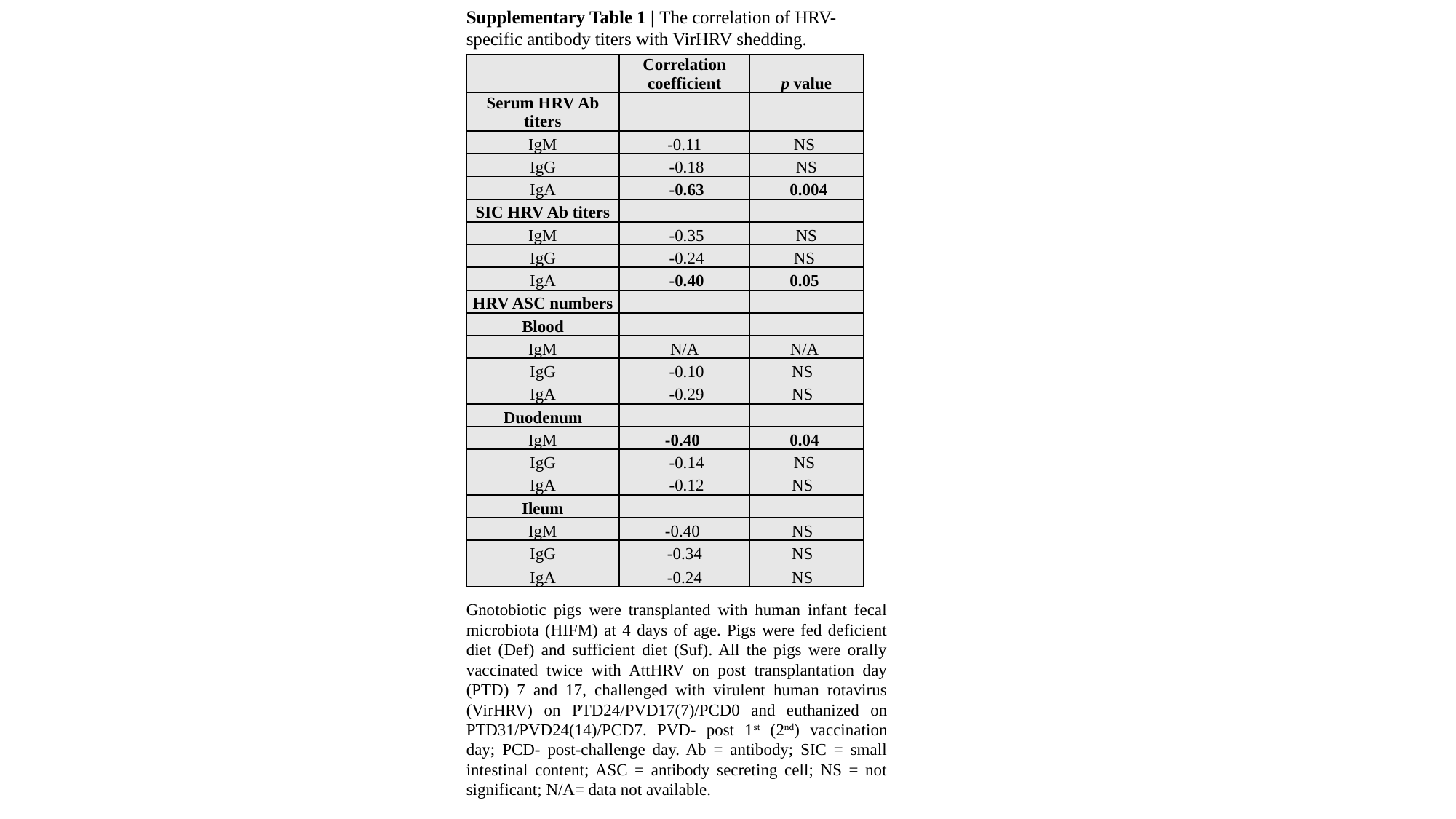

Supplementary Table 1 | The correlation of HRV-specific antibody titers with VirHRV shedding.
| | Correlation coefficient | p value |
| --- | --- | --- |
| Serum HRV Ab titers | | |
| IgM | -0.11 | NS |
| IgG | -0.18 | NS |
| IgA | -0.63 | 0.004 |
| SIC HRV Ab titers | | |
| IgM | -0.35 | NS |
| IgG | -0.24 | NS |
| IgA | -0.40 | 0.05 |
| HRV ASC numbers | | |
| Blood | | |
| IgM | N/A | N/A |
| IgG | -0.10 | NS |
| IgA | -0.29 | NS |
| Duodenum | | |
| IgM | -0.40 | 0.04 |
| IgG | -0.14 | NS |
| IgA | -0.12 | NS |
| Ileum | | |
| IgM | -0.40 | NS |
| IgG | -0.34 | NS |
| IgA | -0.24 | NS |
Gnotobiotic pigs were transplanted with human infant fecal microbiota (HIFM) at 4 days of age. Pigs were fed deficient diet (Def) and sufficient diet (Suf). All the pigs were orally vaccinated twice with AttHRV on post transplantation day (PTD) 7 and 17, challenged with virulent human rotavirus (VirHRV) on PTD24/PVD17(7)/PCD0 and euthanized on PTD31/PVD24(14)/PCD7. PVD- post 1st (2nd) vaccination day; PCD- post-challenge day. Ab = antibody; SIC = small intestinal content; ASC = antibody secreting cell; NS = not significant; N/A= data not available.

## Slide 2
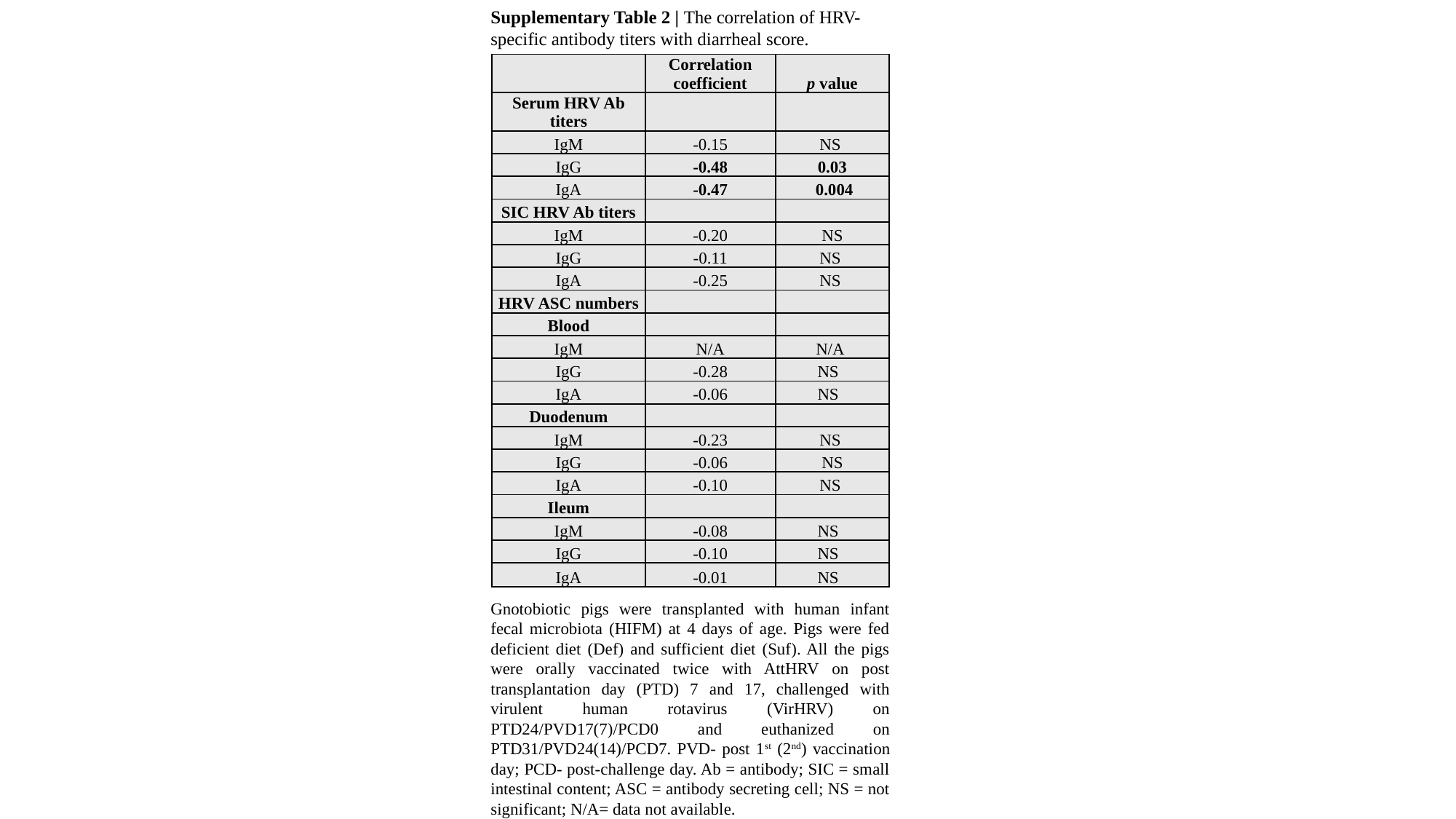

Supplementary Table 2 | The correlation of HRV-specific antibody titers with diarrheal score.
| | Correlation coefficient | p value |
| --- | --- | --- |
| Serum HRV Ab titers | | |
| IgM | -0.15 | NS |
| IgG | -0.48 | 0.03 |
| IgA | -0.47 | 0.004 |
| SIC HRV Ab titers | | |
| IgM | -0.20 | NS |
| IgG | -0.11 | NS |
| IgA | -0.25 | NS |
| HRV ASC numbers | | |
| Blood | | |
| IgM | N/A | N/A |
| IgG | -0.28 | NS |
| IgA | -0.06 | NS |
| Duodenum | | |
| IgM | -0.23 | NS |
| IgG | -0.06 | NS |
| IgA | -0.10 | NS |
| Ileum | | |
| IgM | -0.08 | NS |
| IgG | -0.10 | NS |
| IgA | -0.01 | NS |
Gnotobiotic pigs were transplanted with human infant fecal microbiota (HIFM) at 4 days of age. Pigs were fed deficient diet (Def) and sufficient diet (Suf). All the pigs were orally vaccinated twice with AttHRV on post transplantation day (PTD) 7 and 17, challenged with virulent human rotavirus (VirHRV) on PTD24/PVD17(7)/PCD0 and euthanized on PTD31/PVD24(14)/PCD7. PVD- post 1st (2nd) vaccination day; PCD- post-challenge day. Ab = antibody; SIC = small intestinal content; ASC = antibody secreting cell; NS = not significant; N/A= data not available.

## Slide 3
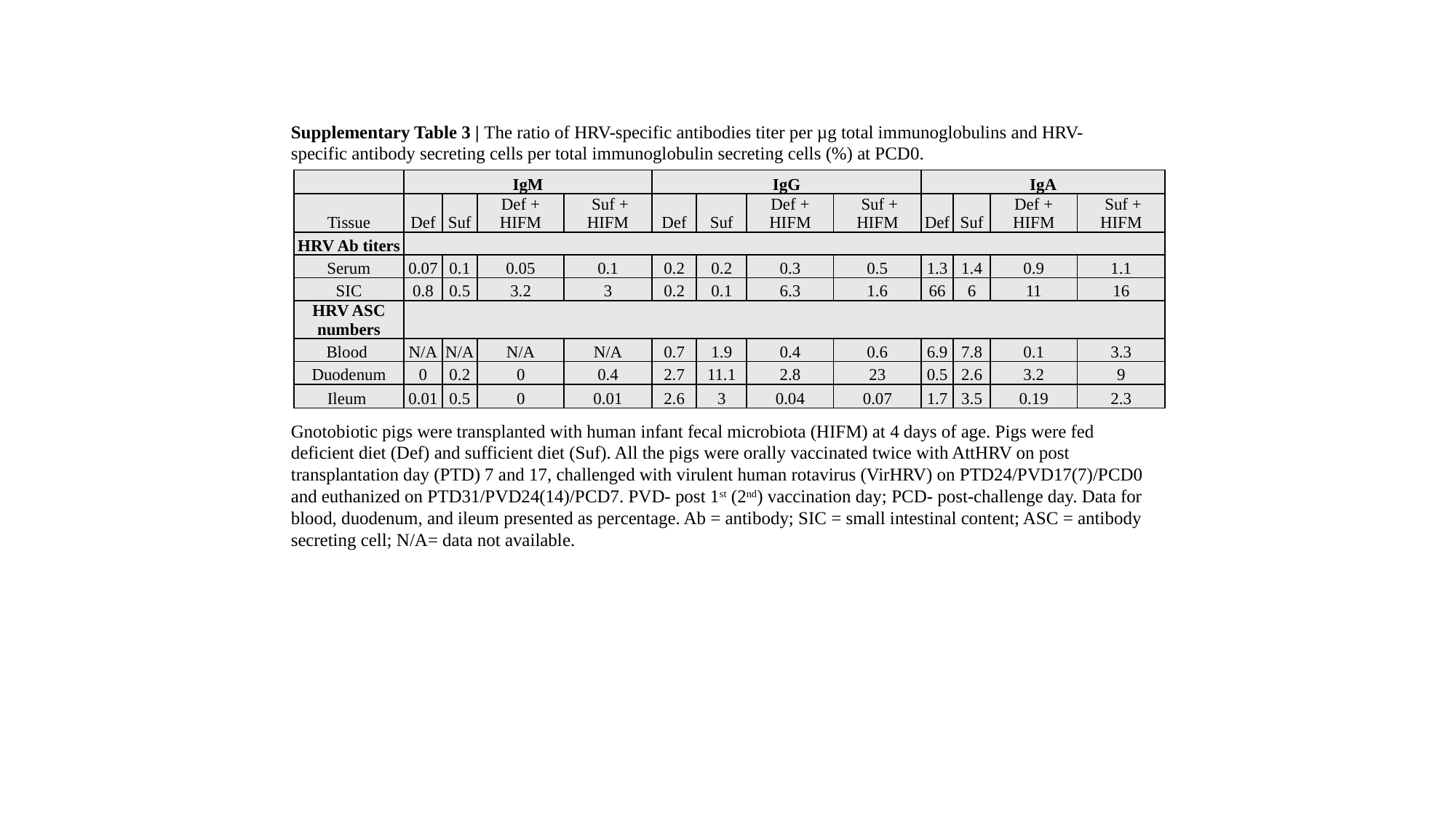

Supplementary Table 3 | The ratio of HRV-specific antibodies titer per µg total immunoglobulins and HRV-specific antibody secreting cells per total immunoglobulin secreting cells (%) at PCD0.
| | IgM | | | | IgG | | | | IgA | | | |
| --- | --- | --- | --- | --- | --- | --- | --- | --- | --- | --- | --- | --- |
| Tissue | Def | Suf | Def + HIFM | Suf + HIFM | Def | Suf | Def + HIFM | Suf + HIFM | Def | Suf | Def + HIFM | Suf + HIFM |
| HRV Ab titers | | | | | | | | | | | | |
| Serum | 0.07 | 0.1 | 0.05 | 0.1 | 0.2 | 0.2 | 0.3 | 0.5 | 1.3 | 1.4 | 0.9 | 1.1 |
| SIC | 0.8 | 0.5 | 3.2 | 3 | 0.2 | 0.1 | 6.3 | 1.6 | 66 | 6 | 11 | 16 |
| HRV ASC numbers | | | | | | | | | | | | |
| Blood | N/A | N/A | N/A | N/A | 0.7 | 1.9 | 0.4 | 0.6 | 6.9 | 7.8 | 0.1 | 3.3 |
| Duodenum | 0 | 0.2 | 0 | 0.4 | 2.7 | 11.1 | 2.8 | 23 | 0.5 | 2.6 | 3.2 | 9 |
| Ileum | 0.01 | 0.5 | 0 | 0.01 | 2.6 | 3 | 0.04 | 0.07 | 1.7 | 3.5 | 0.19 | 2.3 |
Gnotobiotic pigs were transplanted with human infant fecal microbiota (HIFM) at 4 days of age. Pigs were fed deficient diet (Def) and sufficient diet (Suf). All the pigs were orally vaccinated twice with AttHRV on post transplantation day (PTD) 7 and 17, challenged with virulent human rotavirus (VirHRV) on PTD24/PVD17(7)/PCD0 and euthanized on PTD31/PVD24(14)/PCD7. PVD- post 1st (2nd) vaccination day; PCD- post-challenge day. Data for blood, duodenum, and ileum presented as percentage. Ab = antibody; SIC = small intestinal content; ASC = antibody secreting cell; N/A= data not available.

## Slide 4
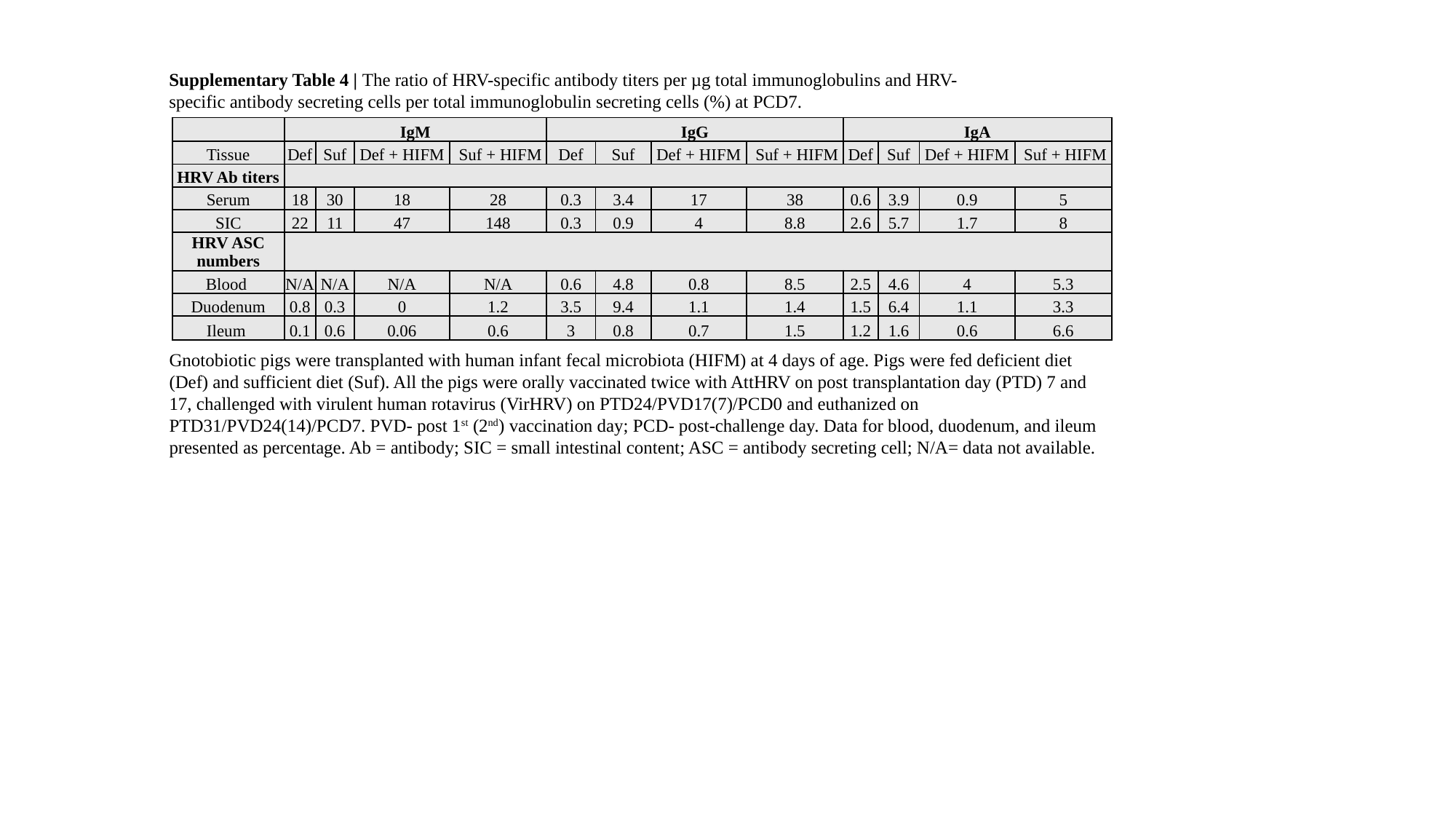

Supplementary Table 4 | The ratio of HRV-specific antibody titers per µg total immunoglobulins and HRV-specific antibody secreting cells per total immunoglobulin secreting cells (%) at PCD7.
| | IgM | | | | IgG | | | | IgA | | | |
| --- | --- | --- | --- | --- | --- | --- | --- | --- | --- | --- | --- | --- |
| Tissue | Def | Suf | Def + HIFM | Suf + HIFM | Def | Suf | Def + HIFM | Suf + HIFM | Def | Suf | Def + HIFM | Suf + HIFM |
| HRV Ab titers | | | | | | | | | | | | |
| Serum | 18 | 30 | 18 | 28 | 0.3 | 3.4 | 17 | 38 | 0.6 | 3.9 | 0.9 | 5 |
| SIC | 22 | 11 | 47 | 148 | 0.3 | 0.9 | 4 | 8.8 | 2.6 | 5.7 | 1.7 | 8 |
| HRV ASC numbers | | | | | | | | | | | | |
| Blood | N/A | N/A | N/A | N/A | 0.6 | 4.8 | 0.8 | 8.5 | 2.5 | 4.6 | 4 | 5.3 |
| Duodenum | 0.8 | 0.3 | 0 | 1.2 | 3.5 | 9.4 | 1.1 | 1.4 | 1.5 | 6.4 | 1.1 | 3.3 |
| Ileum | 0.1 | 0.6 | 0.06 | 0.6 | 3 | 0.8 | 0.7 | 1.5 | 1.2 | 1.6 | 0.6 | 6.6 |
Gnotobiotic pigs were transplanted with human infant fecal microbiota (HIFM) at 4 days of age. Pigs were fed deficient diet (Def) and sufficient diet (Suf). All the pigs were orally vaccinated twice with AttHRV on post transplantation day (PTD) 7 and 17, challenged with virulent human rotavirus (VirHRV) on PTD24/PVD17(7)/PCD0 and euthanized on PTD31/PVD24(14)/PCD7. PVD- post 1st (2nd) vaccination day; PCD- post-challenge day. Data for blood, duodenum, and ileum presented as percentage. Ab = antibody; SIC = small intestinal content; ASC = antibody secreting cell; N/A= data not available.

## Slide 5
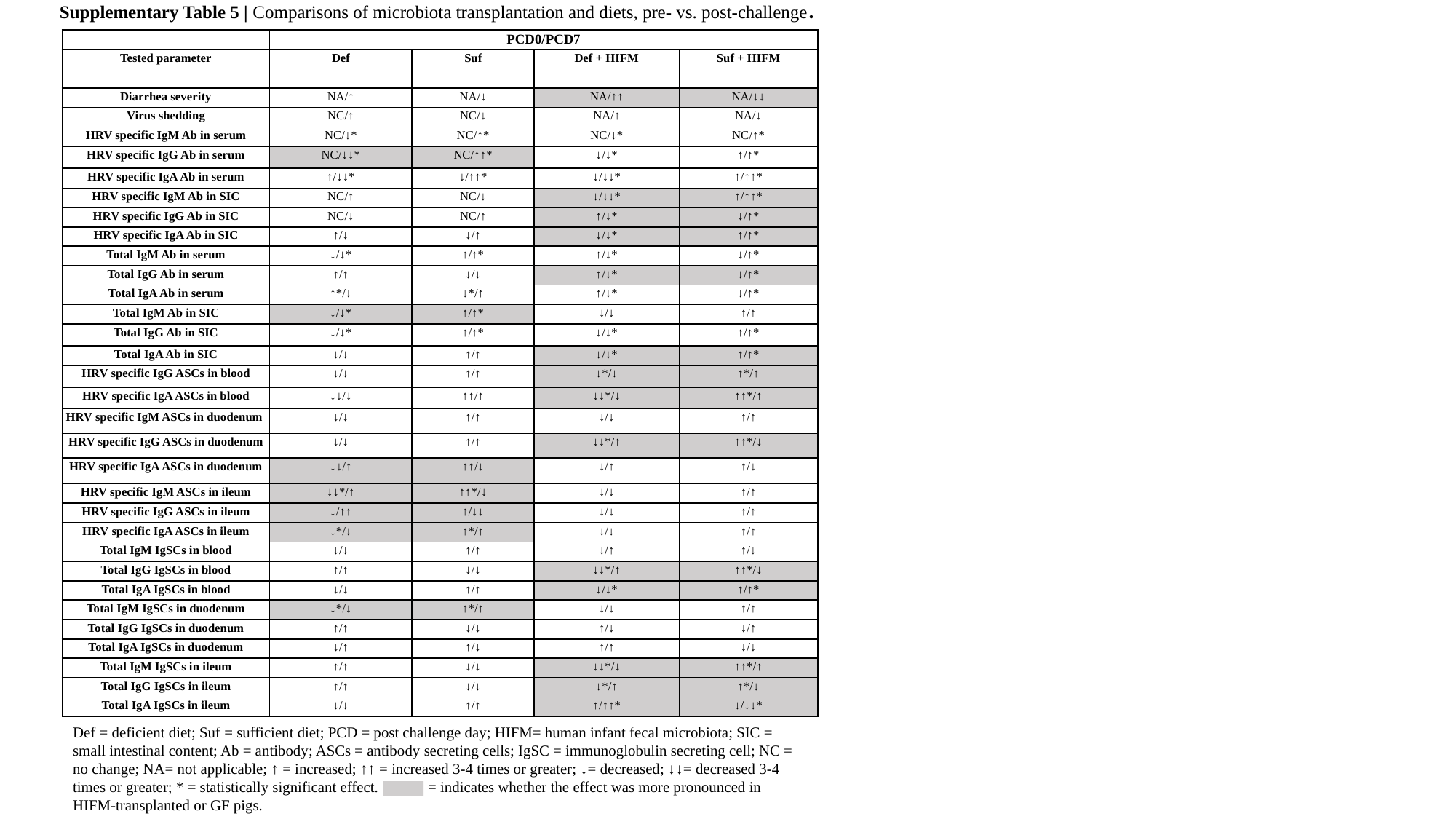

Supplementary Table 5 | Comparisons of microbiota transplantation and diets, pre- vs. post-challenge.
| | PCD0/PCD7 | | | |
| --- | --- | --- | --- | --- |
| Tested parameter | Def | Suf | Def + HIFM | Suf + HIFM |
| Diarrhea severity | NA/↑ | NA/↓ | NA/↑↑ | NA/↓↓ |
| Virus shedding | NC/↑ | NC/↓ | NA/↑ | NA/↓ |
| HRV specific IgM Ab in serum | NC/↓\* | NC/↑\* | NC/↓\* | NC/↑\* |
| HRV specific IgG Ab in serum | NC/↓↓\* | NC/↑↑\* | ↓/↓\* | ↑/↑\* |
| HRV specific IgA Ab in serum | ↑/↓↓\* | ↓/↑↑\* | ↓/↓↓\* | ↑/↑↑\* |
| HRV specific IgM Ab in SIC | NC/↑ | NC/↓ | ↓/↓↓\* | ↑/↑↑\* |
| HRV specific IgG Ab in SIC | NC/↓ | NC/↑ | ↑/↓\* | ↓/↑\* |
| HRV specific IgA Ab in SIC | ↑/↓ | ↓/↑ | ↓/↓\* | ↑/↑\* |
| Total IgM Ab in serum | ↓/↓\* | ↑/↑\* | ↑/↓\* | ↓/↑\* |
| Total IgG Ab in serum | ↑/↑ | ↓/↓ | ↑/↓\* | ↓/↑\* |
| Total IgA Ab in serum | ↑\*/↓ | ↓\*/↑ | ↑/↓\* | ↓/↑\* |
| Total IgM Ab in SIC | ↓/↓\* | ↑/↑\* | ↓/↓ | ↑/↑ |
| Total IgG Ab in SIC | ↓/↓\* | ↑/↑\* | ↓/↓\* | ↑/↑\* |
| Total IgA Ab in SIC | ↓/↓ | ↑/↑ | ↓/↓\* | ↑/↑\* |
| HRV specific IgG ASCs in blood | ↓/↓ | ↑/↑ | ↓\*/↓ | ↑\*/↑ |
| HRV specific IgA ASCs in blood | ↓↓/↓ | ↑↑/↑ | ↓↓\*/↓ | ↑↑\*/↑ |
| HRV specific IgM ASCs in duodenum | ↓/↓ | ↑/↑ | ↓/↓ | ↑/↑ |
| HRV specific IgG ASCs in duodenum | ↓/↓ | ↑/↑ | ↓↓\*/↑ | ↑↑\*/↓ |
| HRV specific IgA ASCs in duodenum | ↓↓/↑ | ↑↑/↓ | ↓/↑ | ↑/↓ |
| HRV specific IgM ASCs in ileum | ↓↓\*/↑ | ↑↑\*/↓ | ↓/↓ | ↑/↑ |
| HRV specific IgG ASCs in ileum | ↓/↑↑ | ↑/↓↓ | ↓/↓ | ↑/↑ |
| HRV specific IgA ASCs in ileum | ↓\*/↓ | ↑\*/↑ | ↓/↓ | ↑/↑ |
| Total IgM IgSCs in blood | ↓/↓ | ↑/↑ | ↓/↑ | ↑/↓ |
| Total IgG IgSCs in blood | ↑/↑ | ↓/↓ | ↓↓\*/↑ | ↑↑\*/↓ |
| Total IgA IgSCs in blood | ↓/↓ | ↑/↑ | ↓/↓\* | ↑/↑\* |
| Total IgM IgSCs in duodenum | ↓\*/↓ | ↑\*/↑ | ↓/↓ | ↑/↑ |
| Total IgG IgSCs in duodenum | ↑/↑ | ↓/↓ | ↑/↓ | ↓/↑ |
| Total IgA IgSCs in duodenum | ↓/↑ | ↑/↓ | ↑/↑ | ↓/↓ |
| Total IgM IgSCs in ileum | ↑/↑ | ↓/↓ | ↓↓\*/↓ | ↑↑\*/↑ |
| Total IgG IgSCs in ileum | ↑/↑ | ↓/↓ | ↓\*/↑ | ↑\*/↓ |
| Total IgA IgSCs in ileum | ↓/↓ | ↑/↑ | ↑/↑↑\* | ↓/↓↓\* |
Def = deficient diet; Suf = sufficient diet; PCD = post challenge day; HIFM= human infant fecal microbiota; SIC = small intestinal content; Ab = antibody; ASCs = antibody secreting cells; IgSC = immunoglobulin secreting cell; NC = no change; NA= not applicable; ↑ = increased; ↑↑ = increased 3-4 times or greater; ↓= decreased; ↓↓= decreased 3-4 times or greater; * = statistically significant effect. = indicates whether the effect was more pronounced in HIFM-transplanted or GF pigs.
